# Comparing the Antimicrobial Actions of Greek Honeys from the Island of Lemnos and Manuka Honey from New Zealand against Clinically Important Bacteria

**DOI:** 10.3390/foods10061402

**Published:** 2021-06-17

**Authors:** Maria Gkoutzouvelidou, Georgios Panos, Maria Nefertiti Xanthou, Alexandros Papachristoforou, Efstathios Giaouris

**Affiliations:** 1 Department of Food Science and Nutrition (DFSN), School of the Environment, University of the Aegean, Ierou Lochou 10 & Makrygianni, 81400 Myrina, Lemnos, Greece; marygktz@hotmail.com (M.G.); fns14088@fns.aegean.gr (G.P.); fns14082@fns.aegean.gr (M.N.X.); alpapach@aegean.gr (A.P.); 2 Department of Agricultural Sciences, Biotechnology and Food Science, Cyprus University of Technology, Archiepiskopou Kyprianou 30, Limassol 3036, Cyprus

**Keywords:** honey, antimicrobial, Lemnos Greece, manuka, bacterial pathogens, minimum inhibitory and bactericidal concentrations, honey quality indexes, melissopalynological analysis

## Abstract

Honey is a natural food with a long history as a traditional medicine because of its many biological characteristics, including antimicrobial, antioxidant, anti-tumor and anti-inflammatory properties. In this study, the antimicrobial actions of eight different honeys from Lemnos island (north-eastern Greece) plus manuka honey (from New Zealand, UMF 30+, licensed in many countries as topical medical preparation) were evaluated against 10 clinically relevant bacteria, including five Gram-positive and five Gram-negative. To achieve this, an agar well diffusion assay measured the diameter of inhibition zones (mm) of two selected concentrations for each honey (25% and 12.5% *v/v*). The minimum inhibitory and bactericidal concentrations (MIC and MBC) of each sample were also calculated and compared against two representative bacterial species (*Salmonella* Typhimurium and *Staphylococcus aureus*) using broth microdilution and agar spot methods, respectively. The pH, water activity (a_w_), 5-hydroxymethylfurfural (HMF) and diastase levels, together with the pollen type and content of each honey, were also determined. Results revealed that all the Lemnos honeys presented antibacterial action, which for some samples was like that of manuka. These all had an acidic pH (3.61 ± 0.04), with a a_w_ ≤ 0.60, while it is worth noting that those found to display the strongest antibacterial actions also presented the lowest HMF content, together with the highest diastase values, both of the latter being used as quality parameters. Pollen composition of the Lemnos honeys was multifloral, underlining the rich plant biodiversity encountered on the island. To summarize, Lemnos honeys could be further exploited as natural antimicrobial systems for use in foods and medicine.

## 1. Introduction

Honey is a natural complex food produced by honeybees (*Apis mellifera* L.) that can be stored for a long time at room temperature without the need to add any preservative. This quality results from the synergistic combination of its low water activity (a_w_ < 0.6), low pH (*ca.* 3.2–4.5) and its antimicrobial compounds, such as hydrogen peroxide (H_2_O_2_), phenolic compounds (such as flavonoids), methylglyoxal (MGO) and antimicrobial peptides (such as bee defensin-1) [1]. Honey has been used for centuries as a nutritional and medicinal product, and the scientific interest for this product has increased significantly in recent years due to its many described biological, preventive and therapeutic properties [2]. Many studies have explored the widely reported antimicrobial action and therapeutic uses of manuka honey, which is native to New Zealand and parts of Australia and is currently licensed in many countries as a topical medical preparation for the treatment of wound infection [3,4]. In recent years, there has been increased interest in the antibacterial properties of honeys produced throughout the world, often in response to the rapid increase of antibiotic-resistant bacteria [5,6] and consumer demand for medicinal foods (nutraceuticals) [7]. In addition, several other bee products, such as propolis, bee pollen (beebread), beeswax, royal jelly and bee venom, have also received increased attention due their promising functional (health-promoting) properties [8,9,10]. Honey properties and taste are known to vary depending on the flora foraged by bees (such as pine, sage, thyme), the geographical foraging area and the local climatic environment (including temperature, soil, rainfall), as well as processing and storage-time conditions [1]. Of note, studies have revealed that the strong antimicrobial actions of some of the tested honeys may be superior to that of manuka [11,12,13], known for its rich MGO content [3]. The craft of beekeeping is very old and still quite popular in Greece, which has the largest per capita consumption of honey in Europe [14]. Greece is also known to produce some of the finest honey in the world. Lemnos is the eighth-biggest island in Greece, is mostly flat and produces exclusively blossom honey (from nectar), which until now has mainly been acquired locally. In this study, the antimicrobial actions of eight honeys produced in different locations of Lemnos island (north-eastern Greece) and that of a manuka honey blend (from New Zealand, UMF 30+) were evaluated against 10 clinically relevant bacteria, including five Gram-positive and five Gram-negative. The pH, water activity, 5-hydroxymethylfurfural (HMF) and diastase levels, together with the pollen type and content of each honey, were also determined. To the best of our knowledge, this is the first report on the physicochemical and antimicrobial properties of Lemnos honey.

## 2. Materials and Methods

### 2.1. Bacterial Strains and Growth Conditions

Ten clinically relevant bacterial strains were used as the target microorganisms. Five were Gram-positive (*Staphylococcus aureus* str. DFSN_B26, *S. epidermidis* str. FMCC_B202, *Enterococcus faecalis* str. ATCC 29212, *Listeria monocytogenes* str. AAL 20074, and *Bacillus cereus* str. ATCC 10876), and five were Gram-negative (*Salmonella enterica* serovar Enteritidis str. P167807, *S. enterica* ser. Typhimurium phage type DT193 str. FMCC_B137, *Escherichia coli* serovar O157:H7 str. ATCC 43888, *Vibrio parahaemolyticus* str. ATCC 17802, and *Pseudomonas aeruginosa* str. ATCC 27853). Before testing, all strains were stored frozen (in cryovials at −80 °C) in Brain Heart Infusion Broth (BHI; Lab M, Heywood, Lancashire, UK) containing 15% (*v/v*) glycerol. Each one was then revivified by streaking a loopful of its frozen suspension on the surface of Tryptone Soy Agar (TSA; Lab M) and incubating it at 37 °C for 24 h (except for *B. cereus*, which was incubated at 30 °C). Working cultures were prepared by inoculating, with a microbiological loop, cells of a well-isolated colony from each preculture into 10 mL of fresh Tryptone Soy Broth (TSB; Lab M) and incubating at 37 °C for 24 h (except for *B. cereus* which was incubated at 30 °C). Both TSA and TSB contained a final sodium chloride (NaCl) concentration of 3% (**w/v**) when they were used for the growth of halophile *V. parahaemolyticus*.

### 2.2. Honey Samples

**Figure 1:**
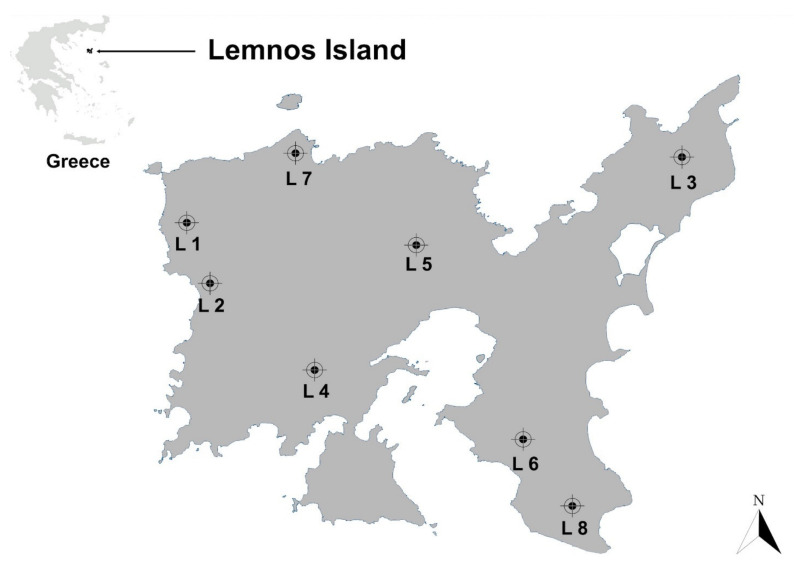
Lemnos island (north-eastern Aegean Sea) from where the eight different honey samples studied in this work were collected. L1–L8: Sampling areas.

Eight freshly produced blossom honeys harvested from beekeepers with apiaries in various locations of Lemnos island (477.6 square kilometers; Figure 1) and one sample of a medical-grade manuka honey blend (UMF 30+; Manuka Health, New Zealand) purchased from a local pharmacy were tested. On arrival at the laboratory in glass vessels, all samples were stored in the dark in a refrigerator, and were analyzed within two months of receipt.

### 2.3. Agar Well Diffusion Assay

Liquid (25 mL) soft TSA medium (i.e., TSB also containing 0.7% *w/v* agar) was initially inoculated with the target microorganism (ca. 10^6^ CFU/mL) and placed in a petri dish (of 90 mm diameter) where it was left to solidify. Eight wells (each with a diameter of 5 mm) were then created in the solidified medium with the help of an inverted Pasteur glass pipette. For each honey sample, two dilutions (25% and 12.5% *v/v*) were prepared using quarter-strength Ringer’s solution (Lab M) as the diluent. Each dilution (40 μL) was then placed in duplicate in the wells prepared in soft TSA, and dishes were left for 2 h at room temperature and finally placed at 37 °C for 24 h (except for *B. cereus*, which was incubated at 30 °C). Soft TSA also contained 3% (*w/v*) NaCl in the case of *V. parahaemolyticus*. Following incubation, the growth inhibition zones around each well were measured with the help of a ruler. Ampicillin (50 μg/μL; Cayman Chemicals, Ann Arbor, MI, USA) and corn glucose syrup (82% *v/v*; Haitoglou Bros SA, Kalochori, Thessaloniki, Greece) were used as positive and negative antimicrobial controls, respectively. The last one was selected because it has the average sugar content of honey [15]. The susceptibility of all the tested strains to the antibiotic had also been verified in preliminary experiments. The experiment was repeated three times using independently grown bacterial cultures.

### 2.4. Determination of Minimum Inhibitory and Bactericidal Concentrations (MIC, MBC) of Each Honey

The MIC of each honey was determined against both *S*. Typhimurium and *S. aureus*, representing Gram-negative and Gram-positive bacteria, using the broth microdilution method, as previously described [16]. Briefly, 10 successive binary dilutions (i.e., 25–0.1% *v/v*) of each honey were prepared using TSB as the diluent. Subsequently, 180 μL of each dilution were transferred to a well (in duplicate) of a sterile flat-bottomed 96-well polystyrene (PS) cell culture plate (transparent, Ref 30096; SPL Life Sciences, Gyeonggi-do, Korea) and 20 μL of a 100-fold dilution of the appropriate bacterial working culture were then added, giving an initial bacterial concentration in each well of ca. 10^5^ CFU/mL. Wells without bacteria and wells without added honey served as negative and positive growth controls, respectively. The plates were sealed with parafilm and statically incubated at 37 °C for 24 h. The growth in each well was turbidimetrically assessed by the naked eye to calculate the MIC value, that is the lowest concentration of each honey that totally inhibited the visible bacterial growth. To calculate MBC, 10 μL from each well showing no visible growth were aspirated and spotted on TSA, and the number of colonies was counted following incubation at 37 °C for 24 h. MBC for each honey was defined as its lowest concentration reducing the initial inoculum by at least three logs (i.e., no appearance of colonies). The experiment was repeated three times using independently grown bacterial cultures.

### 2.5. pH and a_w_ Measurements

The pH of each honey sample was measured using the C931P Consort electrochemical analyzer (Turnhout, Belgium) after mixing 10 g of honey with 75 mL of distilled water, while its a_w_ was determined using the LabTouch instrument Novasina AG (Lachen, Switzerland). Before all measurements were taken, honeys were left outside the refrigerator for sufficient time to reach room temperature, while both instruments were calibrated following the manufacturers’ guidelines. More specifically, the pH meter was calibrated at room temperature using pH buffers 4.01, 7.00 and 10.00 (Mettler Toledo, Columbus, OH, USA), while the 33%, 58% and 73% salinity standards provided by Novasina AG were used for the calibration of a**_w_** instrument, respectively.

### 2.6. Determination of HMF and Diastase Levels

HMF and diastase levels were determined in each honey sample following the Harmonised Methods of the International Honey Commission [17]. More specifically, for the quantification of HMF (mg/kg), the spectrophotometric method of White [18] was applied. To do this, 5 g of each honey sample was diluted in 25 mL of water and transferred into a 50 mL volumetric flask. Then, 0.5 mL of Carrez I solution was added, followed by 0.5 mL of Carrez II solution in the slash, which was filled up to 50 mL with water. The solution was filtered through paper and the first 10 mL of the filtrate was rejected. Aliquots of 5 mL were added to two test tubes, and 5 mL of distilled water was added to one tube (sample solution), while 5 mL of 0.2% (*w/v*) sodium bisulphite solution was added to the second (reference solution). The absorbances of the solutions at 284 and 336 nm were determined using a UV–visible spectrometer (UV-1800, Shimadzu Co., Kyoto, Japan). The HMF content was calculated by the equation HMF (mg/kg) = A**_284_** − A**_336 ×_** 149.7 where A**_284_** is the absorbance at 284 nm, A**_336_** is the absorbance at 336 nm and 149.7 is a factor calculated by the molecular weight of HMF and the mass of the sample.

For the quantification of diastase, the methodology initially described by Schade et al. [19] was followed. To do this, 10 g of each honey sample was placed in a 50 mL beaker in which 5 mL of acetate buffer was added together with 20 mL of water. After samples were dissolved, 3 mL of sodium chloride (0.5 M) were added, and the solution was diluted to 50 mL with water. At the same time, a starch solution was standardized using an iodine solution. Both solutions were warmed at 40 °C. Starch solution (5 mL) was added into 10 mL of honey solution and a stopwatch was started. An aliquot was taken every 5 min and added to 10 mL of iodine solution. The absorbance was recorded and a calibration curve was obtained. According to the Association of Official Analytical Chemists (AOAC) method, the number 300 was divided by the time needed to reach the absorbance value of 0.235 and expressed as Diastase Number (DN) [20]. All chemicals used for these experiments were purchased from Merck KGaA (Darmstadt, Germany).

### 2.7. Determination of the Botanical Origin of Honeys through Melissopalynological Analysis

All honeys were analyzed palynologically using a non-acetalytic technique, according to standard methods [21]. To do this, 10 g of each honey was diluted in 20 mL of distilled water. After centrifuging (at 1690 g for 20 min at room temperature), the sediment was dried at 40 °C and mounted with Entellan on two independent glass microscopic slides. For each replicate, more than 400 pollen grains were counted and digitally photographed using a Motic Compound Microscope B3-223 ASC equipped with a CCD color camera (MoticEurope, S.L.U.; Cabrera de Mar, Barcelona, Spain). These were finally identified with reference to our database pollen grain collection of Lemnos plants, prepared according to standard palynological methods [21], and the results were expressed in percentages. For the palynological analysis of the manuka honey blend, literature sources [22] were used to identify the origin of its digitally photographed pollen grains.

### 2.8. Statistics

All the data of the agar well diffusion assay were analyzed by factorial analysis of variance (ANOVA) followed by Fisher’s post hoc least significant difference (LSD) tests for mean pairwise comparisons to check for any significant differences on the inhibition zone diameters (mm) between the different samples and target bacteria. One-way ANOVA, followed by Fisher’s LSD test, was also applied to discriminate the samples based on their pH. Statistical analyses were done using the software STATISTICA (StatSoft Inc.; Tulsa, OK 74104, USA) and all differences are reported at a significance level of 0.05.

## 3. Results and Discussion

The results of the agar well diffusion assay are presented in Table 1. In general, the Gram-positive bacteria were more resistant compared to the Gram-negative, apart from *P. aeruginosa* for which no inhibition was observed for any of the tested honeys. Similarly, none of the honeys at either tested concentration (i.e., 25% and 12.5% *v/v*) could inhibit the growth of *S. epidermidis*, *L. monocytogenes* and *B. cereus*, while *E. faecalis* was found susceptible only to the action of manuka honey. The two *Salmonella* serovars (i.e., Enteritidis and Typhimurium), *E. coli* O157:H7 and *V. parahaemolyticus,* were inhibited by all nine tested honeys at both concentrations (except for Lemnos honey No. 6, applied at 12.5% against *S*. Enteritidis). *S. aureus* was found susceptible to only two Lemnos honeys (samples No. 7 and No. 8) and to manuka. These two local honey samples, and in particular sample No. 7, were found to present the strongest antibacterial actions, being able to inhibit five of the 10 tested strains. This inhibition against most of the susceptible strains was like that of manuka, which was still able to inhibit 6 of the 10 strains. Glucose syrup, used here as a negative antimicrobial control to test for any inhibition due to possible osmotic effects, was able to inhibit only the four Gram-negative *S*. Enteritidis, *S*. Typhimurium, *E. coli* O157:H7 and *V. parahaemolyticus* strains. However, except against the last halophilic species, the growth inhibition zones of glucose syrup were significantly lower than those recorded following the application of the honey samples. As expected, kanamycin (50 μg/μL), used here as a positive antimicrobial control, was quite effective against all the tested strains, displaying the strongest action against *S*. Enteritidis and the lowest against *P. aeruginosa* (with recorded inhibition zones equal to 35.8 ± 3.2 and 13.3 ± 2.1, respectively).

The MIC and MBC of each honey against *S*. Typhimurium and *S. aureus*, as determined by the broth microdilution and agar spot methods, are presented in Table 2. Lemnos honey No. 2 was found to present the strongest antibacterial action, displaying MIC and MBC against both bacterial species equal to 12.5% (*v/v*). For all the other tested honeys, MIC and MBC were either 25% (*v/v*) or even higher. Glucose syrup could not inhibit either bacteria at the concentrations tested (i.e., 25—0.1% *v/v*). No clear correlation between the antimicrobial results of the two tested methods (i.e., agar-well diffusion and broth microdilution) could be established. Until now, many studies have explored the antimicrobial actions of manuka and other medical-grade honeys, mainly to be used as alternative therapies to treat antibiotic-resistant infections [3,23]. With respect to manuka, this action is known to be related to the value of the Unique Manuka Factor (UMF), which is correlated with the MGO and total phenols content of this honey [3]. On the other hand, Greece has a long history in the craft of beekeeping, and today has the greatest density of bee colonies in Europe [14]. While some studies have been published in the last decade on the antibacterial actions of Greek honeys [11,12,24,25], to the best of our knowledge the current study is the first one reporting the actions of honeys produced in Lemnos. Despite its natural beauty, Lemnos has not yet been overly exposed to high levels of tourism, thus offering an ideal environment for the welfare of bees and the smooth development of beekeeping. In a previous related study investigating the antibacterial actions of 31 Greek and Cypriot honeys against methicillin-resistant *S. aureus* (MRSA) and carbapenem-resistant *P. aeruginosa*, it was shown that the MICs varied between 3.125% (*v/v*) and 25% (*v/v*) depending on the honey sample and target pathogen [12]. In that older study, the MICs of a sample of manuka honey (UMF 25+), which was used as positive control and for comparison, were found equal to 6.25% (*v/v*) and 12.5% (*v/v*) against *S. aureus* and *P. aeruginosa*, respectively.

The pH, a_w_, HMF (mg/kg) and diastase levels (DN), together with the pollen composition (%) of each honey, are shown in Table 3. As expected, the pH values varied between 3.6 (for almost all Lemnos honeys) to 4.3 (for manuka honey). The low pH of honey is an important quality parameter that, together with the low a_w_, assures its microbial stability against all types of microorganisms that could otherwise grow in this product. This low pH is achieved due to the presence of organic acids such as gluconic, citric, succinic, malic, butyric and lactic acid, which are key factors in its taste and color development [26]. Water activity (a_w_) was found to vary from 0.551 (Lemnos honey No. 6) to 0.627 (manuka honey). Water is the second largest component of honey (after sugar), with its percentage varying from 15% to 21% (*v/v*) depending on the botanical origin, level of maturity achieved in the hive and processing and storage conditions [26]. The free fraction of water in any food determines a_w_; in honey this influences physical properties, such as viscosity and crystallization, as well as other important organoleptic parameters determining consumer preference (e.g., color, flavor, solubility). An a_w_ lower than 0.60 protects honey from the growth of osmophilic yeasts that can cause fermentation, which lowers its quality [27]. The pH and a_w_ of glucose syrup measured 4.85 and 0.731, respectively.

HMF is a cyclic aldehyde resulting from sugar degradation through the non-enzymatic Maillard reaction, which is favored under acidic conditions of increased temperature and/or prolonged storage [28]. HMF concentration is generally recognized as an index indicating the freshness of honey, since it tends to increase in aged honey and/or honey processed and/or stored at higher temperatures (denoting the deterioration of the product). Based on a European Union directive, honey available for sale should not contain more than 40 mg/kg of HMF, except for baker’s and tropical honeys, which may have up to 80 mg/kg [29]. HMF levels were here found to range from 10.20 mg/kg (Lemnos honey No. 7) to 28.56 mg/kg (Lemnos honey No. 2). Interestingly, the three honeys found to present the strongest antibacterial actions (through the agar well diffusion assay; i.e., Lemnos honey No. 7, No. 8 and manuka), were also the ones that displayed the lowest HMF concentrations. Notably, these two Lemnos honeys also presented the two highest diastase values (32.09 and 28.55 DN for Lemnos honey No. 7 and No. 8, respectively). Diastases are enzymes (a- and b-amylases) that digest starch to a mixture of maltose and maltotriose. These are naturally present in honey, with their levels varying depending on its floral and geographical origin [26]. Since they are sensitive to heat and may lose their activity during storage, lower levels for those enzymes in honey may indicate overheating during processing (>60 °C) and/or prolonged storage.

Pollen composition of the Lemnos honeys was multifloral, containing pollens from a variety of plant species including myrrh (*Anthillis hermanniae*) and thyme (*Thymus capitatus*)—the dominant pollen grains—plus burdock (*Arctium lappa*), thistle (*Silybum marianum*) and others. This highlights the rich plant biodiversity encountered on the island of Lemnos. Lemnos honey pollens are also derived from plant species that represent important medicinal plants that are still used by locals to treat different ailments [30]. This rich plant biodiversity was not surprising, given that the flora of Lemnos consists of more than 680 plant taxa despite intense human interference (agriculture, stock farming) over almost on the entire island [31].

## 4. Conclusions

In recent years, several studies have been published about the antimicrobial actions of honeys collected from various parts of the world [32,33,34,35,36]. These have revealed promising antimicrobial activity of some honey samples even against multidrug-resistant bacterial pathogens, such as MRSA. These studies also emphasized the variability in the antimicrobial effect of honeys depending on sample and target microorganism, and pointed to the need for further research. Our study, focusing on honeys produced in a Greek island of the north Aegean region (i.e., Lemnos) known for its biodiversity and containing wild plants of medicinal importance such as thyme and myrrh, complements all these other studies. In summary, our results revealed that all the Lemnos honeys presented antibacterial action which, for some samples, was like that of manuka. These all had an acidic pH (3.61 ± 0.04), with an a_w_ ≤ 0.60, while those found to display the strongest antibacterial actions also presented the lowest HMF content, together with the highest diastase values; these latter metrics are traditionally used as quality parameters. Pollen composition of the Lemnos honeys was, as expected, multifloral. Future studies should shed light on the mechanisms underlying this strong action. Such knowledge could assist in the exploitation of these honeys for the development of natural antimicrobial systems that could be used in foods and medicine.

## Figures and Tables

**Table 1:** Diameters (mm) of inhibition zones of honeys, each applied at two concentrations (25% and 12.5% *v/v*) against the target bacteria, as determined by the agar well diffusion assay. Each value represents the mean value ± standard deviation (*n* = 3) and comprises the diameter of the well (5 mm). The inhibition zones of glucose syrup and kanamycin used as negative and positive antimicrobial controls are also indicated. For each separate concentration, mean values sharing at least one common superscript letter are not significantly different (*p* > 0.05). Lower- and capital-case letters are used for the concentrations 25% and 12.5% (*v/v*), respectively. Kanamycin was tested in a sole concentration (50 μg/μL) and its inhibition zones were compared in parallel to each of the two concentrations of all the other samples (nine honey samples plus the glucose syrup).

s/n	Sample	Conc.	Gram−					Gram+				
			*S.* Enterit.	*S.* Typhim.	*E. coli*	*V. parah.*	*P. aerugin.*	*S. aureus*	*S. epiderm.*	*E. faecal.*	*L. monoc.*	*B. cereus*
1	Lemnos honey No. 1	25% (*v/v*)	22.0 ^efghj^ ± 2.0	18.0 ^cd^ ± 0.0	22.0 ^efghj^ ± 0.0	22.0 ^efghj^ ± 1.6	5.0 ^a^ ± 0.0	5.0 ^a^ ± 0.0	5.0 ^a^ ± 0.0	5.0 ^a^ ± 0.0	5.0 ^a^ ± 0.0	5.0 ^a^ ± 0.0
		12.5% (*v/v*)	17.0 ^DEF^ ± 4.2	19.3 ^FGH^ ± 5.8	19.3 ^FGH^ ± 1.2	19.5 ^FGH^ ± 0.7	5.0 ^A^ ± 0.0	5.0 ^A^ ± 0.0	5.0 ^A^ ± 0.0	5.0 ^A^ ± 0.0	5.0 ^A^ ± 0.0	5.0 ^A^ ± 0.0
2	Lemnos honey No. 2	25% (*v/v*)	21.3 ^efgh^ ± 3.1	19.0 ^cde^ ± 1.4	21.3 ^efgh^ ± 3.1	20.7 ^defg^ ± 3.1	5.0 ^a^ ± 0.0	5.0 ^a^ ± 0.0	5.0 ^a^ ± 0.0	5.0 ^a^ ± 0.0	5.0 ^a^ ± 0.0	5.0 ^a^ ± 0.0
		12.5% (*v/v*)	19.0 ^FGH^ ± 1.4	14.5 ^CDE^ ± 5.5	18.7 ^FH^ ± 3.1	19.3 ^FH^ ± 3.2	5.0 ^A^ ± 0.0	5.0 ^A^ ± 0.0	5.0 ^A^ ± 0.0	5.0 ^A^ ± 0.0	5.0 ^A^ ± 0.0	5.0 ^A^ ± 0.0
3	Lemnos honey No. 3	25% (*v/v*)	20.7 ^defg^ ± 1.2	25.0 ^ijklm^ ± 4.2	21.3 ^efgh^ ± 1.2	23.0 ^ghij^ ± 2.6	5.0 ^a^ ± 0.0	5.0 ^a^ ± 0.0	5.0 ^a^ ± 0.0	5.0 ^a^ ± 0.0	5.0 ^a^ ± 0.0	5.0 ^a^ ± 0.0
		12.5% (*v/v*)	17.5 ^DEF^ ± 3.5	9.5 ^B^ ± 3.5	19.3 ^FGH^ ± 1.2	18.3 ^F^ ± 2.1	5.0 ^A^ ± 0.0	5.0 ^A^ ± 0.0	5.0 ^A^ ± 0.0	5.0 ^A^ ± 0.0	5.0 ^A^ ± 0.0	5.0 ^A^ ± 0.0
4	Lemnos honey No. 4	25% (*v/v*)	24.0 ^hijk^ ± 3.5	20.0 ^cdefg^ ± 2.8	21.3 ^efgh^ ± 4.2	20.0 ^cdefg^ ± 2.8	5.0 ^a^ ± 0.0	5.0 ^a^ ± 0.0	5.0 ^a^ ± 0.0	5.0 ^a^ ± 0.0	5.0 ^a^ ± 0.0	5.0 ^a^ ± 0.0
		12.5% (*v/v*)	19.7 ^FGH^ ± 4.5	18.3 ^FH^ ± 2.9	18.3 ^FH^ ± 3.5	18.0 ^EFH^ ± 2.8	5.0 ^A^ ± 0.0	5.0 ^A^ ± 0.0	5.0 ^A^ ± 0.0	5.0 ^A^ ± 0.0	5.0 ^A^ ± 0.0	5.0 ^A^ ± 0.0
5	Lemnos honey No. 5	25% (*v/v*)	23.0 ^fghijk^ ± 1.4	22.0 ^efghj^ ± 2.0	21.3 ^efgh^ ± 4.6	23.0 ^fghijk^ ± 1.4	5.0 ^a^ ± 0.0	5.0 ^a^ ± 0.0	5.0 ^a^ ± 0.0	5.0 ^a^ ± 0.0	5.0 ^a^ ± 0.0	5.0 ^a^ ± 0.0
		12.5% (*v/v*)	20.0 ^FGHI^ ± 0.0	12.0 ^BC^ ± 3.0	21.0 ^FGHIJL^ ± 1.4	21.0 ^FGHIJL^ ± 1.4	5.0 ^A^ ± 0.0	5.0 ^A^ ± 0.0	5.0 ^A^ ± 0.0	5.0 ^A^ ± 0.0	5.0 ^A^ ± 0.0	5.0 ^A^ ± 0.0
6	Lemnos honey No. 6	25% (*v/v*)	20.0 ^cdef^ ± 2.0	23.0 ^fghijk^ ± 4.2	22.0 ^efghj^ ± 2.0	21.0 ^defgh^ ± 1.4	5.0 ^a^ ± 0.0	5.0 ^a^ ± 0.0	5.0 ^a^ ± 0.0	5.0 ^a^ ± 0.0	5.0 ^a^ ± 0.0	5.0 ^a^ ± 0.0
		12.5% (*v/v*)	5.0 ^A^ ± 0.0	12.0 ^BC^ ± 4.2	20.0 ^FGHI^ ± 2.0	18.0 ^EFH^ ± 2.8	5.0 ^A^ ± 0.0	5.0 ^A^ ± 0.0	5.0 ^A^ ± 0.0	5.0 ^A^ ± 0.0	5.0 ^A^ ± 0.0	5.0 ^A^ ± 0.0
7	Lemnos honey No. 7	25% (*v/v*)	27.3 ^lmno^ ± 1.2	22.0 ^efghj^ ± 2.0	28.7 ^nop^ ± 1.2	26.0 ^klmn^ ± 2.0	5.0 ^a^ ± 0.0	30.0 ^op^ ± 0.0	5.0 ^a^ ± 0.0	5.0 ^a^ ± 0.0	5.0 ^a^ ± 0.0	5.0 ^a^ ± 0.0
		12.5% (*v/v*)	22.7 ^GIJKL^ ± 2.3	21.0 ^FGHIJL^ ± 1.4	26.0 ^KMN^ ± 0.0	21.7 ^GHIJL^ ± 5.9	5.0 ^A^ ± 0.0	24.0 ^JKLM^ ± 3.5	5.0 ^A^ ± 0.0	5.0 ^A^ ± 0.0	5.0 ^A^ ± 0.0	5.0 ^A^ ± 0.0
8	Lemnos honey No. 8	25% (*v/v*)	22.0 ^efghj^ ± 2.0	24.7 ^ijkl^ ± 1.2	28.0 ^mno^ ± 0.0	26.0 ^klmn^ ± 2.0	5.0 ^a^ ± 0.0	30.0 ^op^ ± 0.0	5.0 ^a^ ± 0.0	5.0 ^a^ ± 0.0	5.0 ^a^ ± 0.0	5.0 ^a^ ± 0.0
		12.5% (*v/v*)	20.0 ^FGHI^ ± 4.0	20.0 ^FGHI^ ± 0.0	24.7 ^KLM^ ± 1.2	23.3 ^IJKLM^ ± 3.1	5.0 ^A^ ± 0.0	26.7 ^MN^ ± 2.3	5.0 ^A^ ± 0.0	5.0 ^A^ ± 0.0	5.0 ^A^ ± 0.0	5.0 ^A^ ± 0.0
9	Manuka honey	25% (*v/v*)	24.0 ^hijk^ ± 2.0	22.0 ^efghj^ ± 0.0	28.0 ^lmno^ ± 0.0	22.0 ^efghj^ ± 0.0	5.0 ^a^ ± 0.0	30.0 ^op^ ± 3.5	5.0 ^a^ ± 0.0	32.0 ^pq^ ± 5.7	5.0 ^a^ ± 0.0	5.0 ^a^ ± 0.0
		12.5% (*v/v*)	21.0 ^FGHIJL^ ± 1.4	20.0 ^FGHI^ ± 0.0	25.0 ^KLMN^ ± 1.4	20.0 ^FGHI^ ± 0.0	5.0 ^A^ ± 0.0	25.3 ^KMN^ ± 4.2	5.0 ^A^ ± 0.0	29.0 ^N^ ± 4.2	5.0 ^A^ ± 0.0	5.0 ^A^ ± 0.0
10	Glucose syrup (82% *v/v*)	25% (*v/v*)	11.5 ^b^ ± 6.4	12.0 ^b^ ± 4.2	16.5 ^c^ ± 2.1	21.3 ^efgh^ ± 1.0	5.0 ^a^ ± 0.0	5.0 ^a^ ± 0.0	5.0 ^a^ ± 0.0	5.0 ^a^ ± 0.0	5.0 ^a^ ± 0.0	5.0 ^a^ ± 0.0
		12.5% (*v/v*)	10.0 ^B^ ± 0.0	5.0 ^A^ ± 0.0	14.0 ^CD^ ± 3.5	18.7 ^FH^ ± 3.2	5.0 ^A^ ± 0.0	5.0 ^A^ ± 0.0	5.0 ^A^ ± 0.0	5.0 ^A^ ± 0.0	5.0 ^A^ ± 0.0	5.0 ^A^ ± 0.0
11	Kanamycin	50 μg/μL	35.8 ^rP^ ± 3.2	33.9 ^qO^ ± 2.9	35.7 ^rP^ ± 3.2	32.9 ^qO^ ± 3.1	13.3 ^bB^ ± 2.1	24.5 ^ikKM^ ± 2.1	33.7 ^qO^ ± 2.0	21.6 ^efghGIJ^ ± 1.0	25.1 ^iklKM^ ± 2.4	27.7 ^mnoN^ ± 1.6

**Table 2:** MIC and MBC of each honey against *S*. Typhimurium and *S. aureus* as determined by the broth microdilution and agar spot methods. The MIC and MBC of glucose syrup (82% *v/v*) used as a negative antimicrobial control are also indicated.

s/n	Sample	MIC		MBC	
		*S.* Typhimurium	*S. aureus*	*S.* Typhimurium	*S. aureus*
1	Lemnos honey No. 1	>25%	>25%	>25%	>25%
2	Lemnos honey No. 2	12.50%	12.50%	12.50%	12.50%
3	Lemnos honey No. 3	>25%	>25%	>25%	>25%
4	Lemnos honey No. 4	25%	25%	25%	25%
5	Lemnos honey No. 5	>25%	>25%	>25%	>25%
6	Lemnos honey No. 6	>25%	25%	>25%	25%
7	Lemnos honey No. 7	25%	25%	25%	25%
8	Lemnos honey No. 8	25%	25%	25%	25%
9	Manuka honey	>25%	25%	>25%	25%
10	Glucose syrup (82% *v/v*)	>25%	>25%	>25%	>25%

**Table 3:** pH, a_w_, HMF (mg/kg), diastase (DN) and pollen composition (%) of each honey. The pH and a_w_ of glucose syrup (82% *v/v*) are also indicated. For pH, mean values (*n* = 3) sharing at least one common superscript letter are not significantly different (*p* > 0.05).

s/n	Sample	pH	a_w_	HMF (mg/kg)	Diastase (DN)	Dominant Pollen Grains Composition (%)
1	Lemnos honey No. 1	3.55 ^a^ ± 0.00	0.574	25.71	15.45	*Antillis hermanniae* 48.3%; *Sinapis arvensis* 12.1%; *Melia azedarah* 8.7%; *Thymus capitatus* 2.5%
2	Lemnos honey No. 2	3.61 ^b^ ± 0.02	0.587	28.56	14.61	*Antillis hermanniae* 29.1%; *Arctium lappa* 13.7%; *Thymus capitatus* 4.2%; *Melia azedarah* 4.2%; *Ferula communis* 1/3%
3	Lemnos honey No. 3	3.60 ^b^ ± 0.03	0.568	25.08	15.90	*Echium vulgare* 33.0%; *Antillis hermanniae* 23.0%; *Pyrus amigdaliformis* 11.0%; *Melia azedarah* 8.0; *Arctium lappa* 7.5%; *Thymus capitatus* 1.5%
4	Lemnos honey No. 4	3.62 ^b^ ± 0.02	0.574	19.22	18.79	*Antillis hermanniae* 25.3%; *Echium vulgare* 18.4%; *Sinapis arvensis* 16.3%; *Melia azedarah* 8.6%; *Arctium lappa* 5.3%; *Thymus capitatus* 2.5%
5	Lemnos honey No. 5	3.60 ^b^ ± 0.02	0.597	20.74	20.11	*Rubus fruticosus* 11.9%; *Pyrus amigdaliformis* 8.6%; *Thymus capitatus* 4.8%; *Echium vulgare* 3.3%; *Melia azedarah* 1.9%; *Antillis hermanniae* 1.0%
6	Lemnos honey No. 6	3.67 ^c^ ± 0.01	0.551	24.52	13.60	*Echium vulgare* 18.3%; *Antillis hermanniae* 10.2%; *Pyrus amigdaliformis* 8.8%; *Arctium lappa* 7.3%; *Rubus fruticosus* 6.8%; *Thymus capitatus* 6.8%; *Melia azedarah* 5.9%; *Silybum marianum* 3.1%
7	Lemnos honey No. 7	3.62 ^b^ ± 0.03	0.570	10.20	32.09	*Thymus capitatus* 23.3%; *Melia azedarah* 7.0%; *Rubus fruticosus* 7.0%; *Antillis hermanniae* 5.8%; *Silybum marianum* 3.5%; *Hypericum perforatum* 3.5%
8	Lemnos honey No. 8	3.63 ^b^ ± 0.02	0.604	15.47	28.55	*Echium vulgare* 19.5%; *Antillis hermanniae* 13.7%; *Rubus fruticosus* 12.7%. *Thymus capitatus* 10.2%; *Pyrus amigdaliformis* 9.3%
9	Manuka honey	4.26 ^d^ ± 0.03	0.627	16.42	19.03	*Leptospermum scoparium* 75.8%, *Trifolium repens*, 14.2%, Lotus type 9.2%
10	Glucose syrup (82% *v/v*)	4.85 ^e^ ± 0.03	0.731	-	-	-

## Data Availability

The data presented in this study are available on request from the corresponding author.
